# Modeling and Predicting the Stress Relaxation of Composites with Short and Randomly Oriented Fibers

**DOI:** 10.3390/ma10101207

**Published:** 2017-10-20

**Authors:** Numaira Obaid, Mark T. Kortschot, Mohini Sain

**Affiliations:** 1 Advanced Materials Group, Department of Chemical Engineering and Applied Chemistry, University of Toronto, Toronto, ON M5S 3E5, Canada; numaira.obaid@mail.utoronto.ca; 2 Centre for Biocomposites and Biomaterial Processing, Faculty of Forestry, University of Toronto, Toronto, ON M5S 3B3, Canada; m.sain@utoronto.ca

**Keywords:** stress relaxation, viscoelasticity, composites, random orientation, simulations

## Abstract

The addition of short fibers has been experimentally observed to slow the stress relaxation of viscoelastic polymers, producing a change in the relaxation time constant. Our recent study attributed this effect of fibers on stress relaxation behavior to the interfacial shear stress transfer at the fiber-matrix interface. This model explained the effect of fiber addition on stress relaxation without the need to postulate structural changes at the interface. In our previous study, we developed an analytical model for the effect of fully aligned short fibers, and the model predictions were successfully compared to finite element simulations. However, in most industrial applications of short-fiber composites, fibers are not aligned, and hence it is necessary to examine the time dependence of viscoelastic polymers containing randomly oriented short fibers. In this study, we propose an analytical model to predict the stress relaxation behavior of short-fiber composites where the fibers are randomly oriented. The model predictions were compared to results obtained from Monte Carlo finite element simulations, and good agreement between the two was observed. The analytical model provides an excellent tool to accurately predict the stress relaxation behavior of randomly oriented short-fiber composites.

## 1. Introduction

The high modulus of short-fiber-reinforced polymers has made them useful in demanding load-bearing applications such as high-performance sporting equipment. However, because of the inherent viscoelasticity of the matrix phase, polymer composites are prone to creep [1] and stress relaxation, making it a challenge when considering composites for long-term applications. A better understanding of composite viscoelasticity is needed so that long-term behavior can be better predicted.

A stress relaxation test, where a constant strain is applied to a specimen and the decay of stress is monitored, is a straightforward way of characterizing polymer viscoelasticity. Since viscoelastic properties all result from the same molecular mechanisms, a model for stress relaxation in composites would also shed light on creep or dynamic mechanical behavior. 

The interaction between the fiber and matrix in a short-fiber composite is quite complex, and it has been a challenge to understand the effect of fibers on the viscoelastic properties of these materials. In particular, the effect of fibers on stress relaxation behavior is still not well-understood. Purely elastic fibers should, of course, increase the elastic component (i.e., increase the absolute stiffness), but would not be expected to alter the time-dependent behavior of the material, which is traditionally attributed to the viscoelastic matrix. However, in many studies, researchers have found that the addition of short fibers to a polymer slows the stress-relaxation process. Kutty and Nando [2] investigated the effect of short Kevlar fibers on polyurethanes and found that increasing the fiber content resulted in slower stress relaxation rates. Suhara, Kutty, and Nando [3] showed that increasing the content of short polyester fibers decreased the stress relaxation rate in polyurethanes. The same effects were observed by Saeed et al. [4] and Sreekala et al. [5] for the effect of glass fibers embedded in different matrices.

Two main mechanisms have been proposed in the literature to explain the change in the time constant for stress relaxation with the addition of elastic fibers. The first explanation is that since stress relaxation occurs by rearrangement of the secondary bonds in a polymer, the physical presence of fibers impedes molecular rearrangement of the polymer near the interface, resulting in a slower relaxation of the matrix. Considering the scale at which the molecular rearrangements of secondary bonds occur (angstroms), it seems unlikely that the volume of polymer close enough to a fiber surface to be affected could result in significant changes to the bulk properties of the composite. 

The second theory proposed in the literature focuses on chemical bonding at the fiber/matrix interface. These studies propose that when a fiber is added into a matrix, strong covalent bonds are formed at the interface. During stress relaxation, these additional covalent bonds must be overcome to allow polymer mobility and stress relaxation. Due to these additional bonds, the stress relaxation rates are expected to be slower. George et al. [6], and later Geethamma et al. [7] and Mirzaei et al. [8], have all suggested this mechanism.

Experimental studies agree that the presence of elastic fibers can slow the stress relaxation rate of polymers in which they are embedded. Both of the previously proposed mechanisms rely on speculation regarding the molecular interactions at the fiber-matrix interface. Furthermore, they do not provide any quantitative predictions of the changes in stress relaxation due to fiber addition.

Short-fiber composite viscoelasticity has also been extensively modeled using a tensor elasticity approach to compute the stress field around elastic inclusions embedded in viscoelastic matrices [9,10]. Unfortunately, the mathematical complexity of these formulations makes it very challenging to make predictions of viscoelasticity based on measurable material properties. While some authors have compared their results to experimental data [11], this is usually very limited. 

In a previous paper, we proposed a novel explanation for the effect of elastic fibers on the time-dependency of a polymer matrix composite [12]. We developed a quantitative model based on simple composite micromechanics, by explicitly accounting for the time-dependent shear stress transfer at the fiber-matrix interface. In contrast to previous theories, which were primarily focused on attributing the effect of fibers to changes in the structure near the fiber interface, our model can predict stress relaxation without postulating such changes. 

In our previous study, we specifically examined the effect of short fibers fully aligned with the load direction on composite stress relaxation. In that study, an analytical model was developed, studied parametrically, and then validated by finite element experiments. The analytical model showed that the time-dependence of polymer composites was influenced by two key factors. Firstly, the tensile modulus of the matrix is, of course, time-dependent. However, equally important is its time-dependent shear modulus, which results in a time-dependent shear stress transfer to the fiber. This causes the stress carried by the fiber to also decay with time, causing the overall time-dependence of the composite to be non-linear upon the addition of elastic fibers.

In this study, we extend the model to account for random fiber orientation and validate it using a Monte Carlo finite element simulation. A Monte Carlo approach is one in which results are obtained and conclusions are made based on repeated random sampling. In this study, five replicates were conducted at each fiber content. For each replicate, fibers with random positions and orientations were generated to ensure that the results accurately represented a truly random system. These individual replicates were averaged to understand the effect of fiber content. Thus, for this study, the Monte Carlo approach was used to determine the effect of fiber content by conducting repeated random sampling. Since these simulations incorporate the inherent randomness of fiber positions and orientations, by adopting this approach these simulations can be considered equivalent to lab-scale experiments. 

We also use the model to understand critical differences in the expected viscoelastic behavior of aligned versus misaligned fiber composites.

## 2. Analytical Model

The analytical model proposed previously was based on a simple shear-lag model, originally developed by Cox [13]. While this model has been widely used to predict the elastic properties of short-fiber composites, it had not previously been used to model stress relaxation. For polymer composites containing short fibers aligned in the load direction, the stress relaxation modulus was derived in Reference [12] and the result of this derivation is expressed in Equations (1) and (2).

(1) $$E_{c}\left(t\right)=V_{m}\left[E_{\infty }+\left(E_{0}-E_{\infty }\right)\exp\left(-\frac{t}{\tau }\right)\right]+V_{f}E_{f\;}\left(1-\frac{tanh\left(n\left(t\right)s\right)}{n\left(t\right)s}\right)$$

(2) $$n\left(t\right)={\left[\frac{4}{E_{f}\ln\left(\frac{P_{f}}{V_{f}}\right)}\right]}^{\frac{1}{2}}{\left[G_{\infty }+\left(G_{0}-G_{\infty }\right)\exp\left(-\frac{t}{\tau }\right)\right]}^{\frac{1}{2}}$$

Equation (1) is a modified rule of mixtures equation for the modulus of composites containing short fibers with an aspect ratio (the length-to-diameter ratio of the fiber) of *s*. The first term represents the matrix contribution scaled by its volume fraction ($V_{m}$). The matrix modulus is allowed to decay with time (*t*), as it must in a polymer matrix composite. The relaxation of the matrix modulus is modeled using a one-component Prony Series consisting of the instantaneous modulus ($E_{0}$), long-term modulus ($E_{\infty }$), and the relaxation time constant (τ). The second term accounts for the contribution of the fibers, where $V_{f}E_{f}$ (the product of the fiber volume fraction and fiber modulus) is reduced by the so-called “shear-lag” factor to account for the fact that the short fibers are not fully loaded along their length. The shear-lag factor (n(t)) is determined by Equation (2), in keeping with the original derivation by Cox which consisted of fiber characteristics such as their packing factor ($P_{f}$), fiber volume fraction, and fiber modulus, as well as the shear modulus of the matrix. However, in this analytical model, the shear modulus of the matrix is allowed to decay with time. The critical effect of this decay in shear modulus was overlooked in previous studies of polymer composite viscoelasticity. The equation assumed the polymer to be an isotropic material, and thus, the elastic and shear moduli were related via Poisson’s ratio and the decay in the matrix shear modulus also followed a one-component Prony Series analogous to that of the elastic modulus.

Equations (1) and (2) assume that the stress relaxation modulus decays following a Prony Series, and that the rate of relaxation is characterized by a relaxation rate constant (τ). τ represents the time required for the modulus to drop to a fraction of $\frac{1}{e}$ of its initial value during a stress relaxation test. Previous studies have shown that increasing the fiber content resulted in an increase in the relaxation rate constant, representing a change in the effective time-dependency of the material. Our previous paper showed that the change in relaxation rate constant can be predicted quite well by incorporating time-dependent shear stress transfer at the fiber-matrix interface.

In many practical applications, especially those that utilize short fibers, fibers are not highly aligned in the load direction. The misorientation of fibers changes the overall stress distribution in a composite, and it is therefore important to develop an accurate analytical model to predict the stress relaxation behavior of composites with misoriented fibers. 

**Figure 1:**
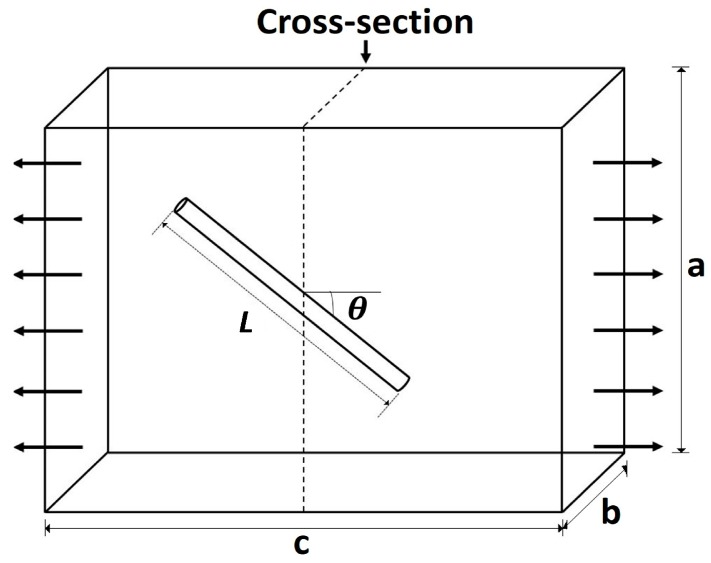
The load carried by a fiber in the loading axis can be calculated through a cross-line perpendicular to the loading direction.

The model for the elastic modulus of a short-fiber composite with misoriented fibers was developed in the original paper by Cox [14], but here we will use the derivation presented by Jayaraman and Kortschot for fibers that are randomly oriented in the plane of the specimen [15]. The model considers a fiber of length *L* and cross-sectional area $A_{f}=\pi r_{f}^{2}$ embedded within a matrix and misoriented at an angle *θ* from the direction of loading, as shown in Figure 1. An imaginary cross-section, perpendicular to the loading direction, is used to assess the total contribution of the fiber to the stress in the composite normal to the cross-section. 

It is assumed that the fibers have a distribution in orientation with probability density functions, as defined in Equation (3), respectively.

(3) $$\int_{0}^{\pi /2}g\left(\theta \right)d\theta =1$$

Because the fiber is not oriented in the loading direction, the projected length of the fiber in the loading direction is then:

(4) $$L_{x}=Lcos\theta$$

The volume fraction of the fibers, taking into account the total number of fibers (N) and their individual fiber volumes (area of the fiber ($A_{f}$) multiplied by their length), is calculated as:

(5) $$V_{f}=\frac{Total\;Volume\;of\;Fibers}{Total\;Volume\;of\;Specimen}=\frac{NA_{f}L}{abc}$$

This equation can be rewritten to calculate the total number of fibers in the specimen as follows:

(6) $$N=\frac{V_{f}abc}{A_{f}L}$$

The number of fibers with an orientation between (θ+dθ) is calculated using its probability density function:

(7) $$N_{\theta }=\left[\frac{V_{f}abc}{A_{f}L}\right]\left(g\left(\theta \right)d\theta \right)$$

The total length of fibers oriented in the θ direction is calculated by considering the projected length of these fibers along the loading direction (from Equation (4)):

(8) $$L_{T}=N_{\theta }L_{x}$$

(9) $$L_{T}=\left[\frac{V_{f}abc}{A_{f}L}\right]g\left(\theta \right)d\theta \left(Lcos\theta \right)$$

Now, we can reconsider the concept of the imaginary cross-section to assess the total load carried by the fibers. The number of fibers intersecting a cross-section can be simplified as the total projected length of the fibers in the loading axis divided by the total length of the specimen in the loading direction.

(10) $$N_{SCAN}=\frac{L_{T}}{c}$$

The total load carried by all fibers in the cross-section is:

(11) $$F_{T}=\underset{\theta }{\sum }N_{SCAN}F_{x}$$

$F_{x}$ has been developed previously as [16]:

(12) $$F_{x}=\bar{F_{f}}cos\theta =\emptyset A_{f}E_{f}\varepsilon_{0}\left({cos}^{2}\theta -v_{s}{sin}^{2}\theta cos\theta \right)$$

where $v_{s}$ is the Poisson’s ratio of the material, $\varepsilon_{0}$ is the strain in the material, and ∅ is the shear-lag factor defined by Cox [10]. Thus,

(13) $$F_{T}=\int_{0}^{\pi /2}\emptyset A_{f}E_{f}\varepsilon_{0}\left({cos}^{2}\theta -v_{s}{sin}^{2}\theta cos\theta \right)\left[\left[\frac{V_{f}ab}{A_{f}L}\right]g\left(\theta \right)d\theta \right]$$

The integral can be solved as shown below with an orientation-based factor ($C_{PP}$):

(14) $$F_{T}=V_{f}E_{f}ab\varepsilon_{0}\left(C_{PP}\right)$$

(15) $$C_{PP}=\int_{0}^{\infty }g\left(\theta \right)(\cos^{4}\theta -v_{s}\sin^{2}\theta \cos^{2}\theta )d\theta$$

If the model is evaluated considering a truly random orientation in the fibers, then there is equal probability that the fiber has a misorientation angle (θ) between 0 and π/2 :

(16) $$g\left(\theta \right)=\frac{1}{\pi /2}\;\mathrm{if}\;0<\theta \leq \pi /2$$

(17) g(θ)=0 if θ> π/2

If g(θ)=2/π, the orientation-based factor further simplifies to:

(18) $$C_{PP}=\frac{2}{\pi }\;\left(\frac{3}{8}\right)\left(\frac{\pi }{2}\right)-\frac{2}{\pi }\;v_{s}\left(\frac{\pi }{16}\right)$$

The Poisson’s ratio of a typical polymeric material is $v_{s}=1/3$; however, during stress relaxation, the polymer relaxes and its Poisson’s ratio approaches $v_{s}=1/2$ [17]. Using $v_{s}=1/2$, the orientation-based factor simplifies to:

(19) $$C_{PP}=\frac{5}{16}$$

Equation (14) can be simplified to:

(20) $$F_{T}=V_{f}E_{f}ab\varepsilon_{0}\left(\frac{5}{16}\right)$$

The contribution by the fibers is therefore reduced by a factor of 5/16  when the fibers are randomly oriented in a plane. The elastic modulus of such a composite must be similarly reduced, and thus, Cox’s shear lag prediction can be modified to incorporate this shrinkage:

(21) $$E_{c}=\frac{5V_{f}E_{f\;}}{16}\left(1-\frac{tanh\left(ns\right)}{ns}\right)+V_{m}E_{m}$$

The following model is derived for the stress relaxation behavior of composites consisting of a viscoelastic matrix embedded with short, elastic fibers oriented randomly in the plane. The analytical model takes into account the time-dependent shear modulus of the matrix in addition to its time-dependent elastic modulus.

(22) $$E_{c}=\frac{5V_{f}E_{f\;}}{16}\left(1-\frac{tanh\left(n\left(t\right)s\right)}{n\left(t\right)s}\right)+V_{m}E_{m}\left(t\right)$$

(23) $$n\left(t\right)={\left[\frac{4}{E_{f}\ln\left(\frac{P_{f}}{V_{f}}\right)}\right]}^{\frac{1}{2}}{\left[G_{m}\left(t\right)\right]}^{\frac{1}{2}}.$$

Note that $E_{m}$ and $G_{m}$. are both functions of *t*, but that $E_{f}$. is a fixed quantity for glass fibers. The model can be used to predict that, in the case of randomly oriented fibers, the time-dependent effectiveness factor is scaled by a factor of 5/16, reducing the effect of fibers on the overall stress relaxation behavior when compared to their oriented counterparts. Thus, the model shows that, unlike oriented composites, where time-dependence is influenced by both the shear and tensile moduli of the matrix, the time-dependence of misoriented composites is primarily influenced by the tensile modulus of the matrix and the effect of the time-dependent shear modulus is comparatively lower. We would therefore expect a more subtle relationship between fiber volume fraction and the relaxation time constant in randomly oriented fibers.

## 3. Monte Carlo Finite Element Simulations

Finite Element (FE) experiments were conducted in Abaqus/CAE (version 6.16, Simulia, Dassault Systemes, Paris, France) and were compared to the predictions from the analytical model to determine its accuracy. In these FE experiments, the matrix was defined as a viscoelastic material having an instantaneous modulus of 1 GPa, a long-term modulus of 0.5 GPa, a relaxation-time constant of 100 s, and a Poisson’s ratio of 0.5. The fibers were defined as having an elastic modulus of 80 GPa, a Poisson’s ratio of 0.2, a length of 260 μm, and an aspect ratio of 16. 

The model consisted of a two-phase system including a matrix and fibers. A rigid body was used to apply boundary conditions that corresponded to a stress relaxation experiment. A tie constraint was applied between the rigid body and the matrix surfaces. The matrix was defined as a three-dimensional deformable object while the fibers were defined as one-dimensional beam elements for computational efficiency. The interaction between the two phases was defined by defining the fibers as being embedded in the matrix with perfect bonding. High mesh densities were applied to both the matrix and the fiber: the matrix mesh consisted of a total of 2211 quadratic elements of type C3D20R while the fiber mesh consisted of 40 quadratic elements of type B32 per fiber. 

The experiments were conducted at various fiber contents and the number of fibers was altered in accordance with the desired fiber volume fraction. Five replicate simulations were conducted for each fiber content. For each replicate, a Python script was used to assign a random position and random orientation to each fiber. This ensured that each new experiment contained fiber placements that were random and different from previous replicates. The mesh was autogenerated on the edges of the fiber.

The finite element experiment consisted of two analysis steps: An instantaneous strain was applied, followed by 400 s during which stress decay was observed. Default solver settings were used in both analysis steps. The modulus of the composite was determined using the cross-sectional area, the applied force, and displacement of the rigid body. The data was collected at 10 s intervals with a minimum increment time step of 0.004 s.

## 4. Results

**Figure 2:**
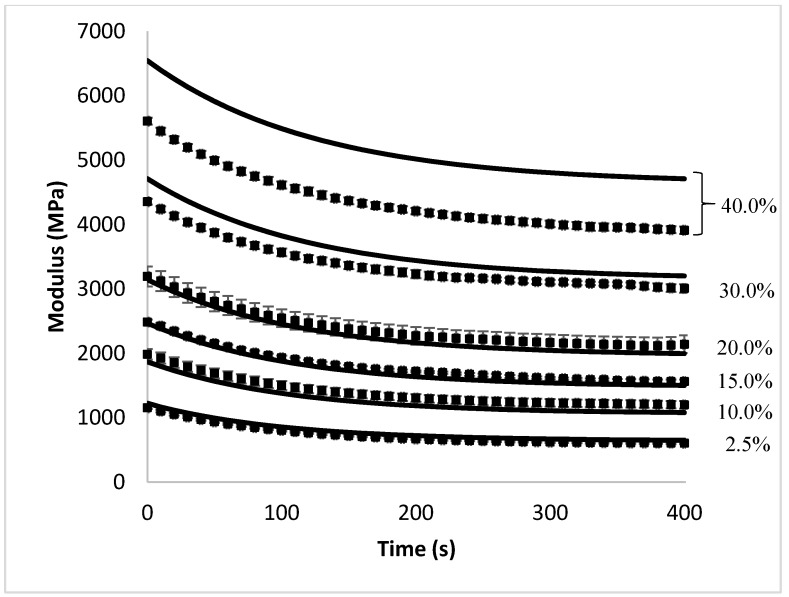
A comparison of the overall stress relaxation profile of short-fiber composites shows excellent agreement between the predictions of the analytical model (-) and the results obtained from the finite element experiments (■).

**Figure 3:**
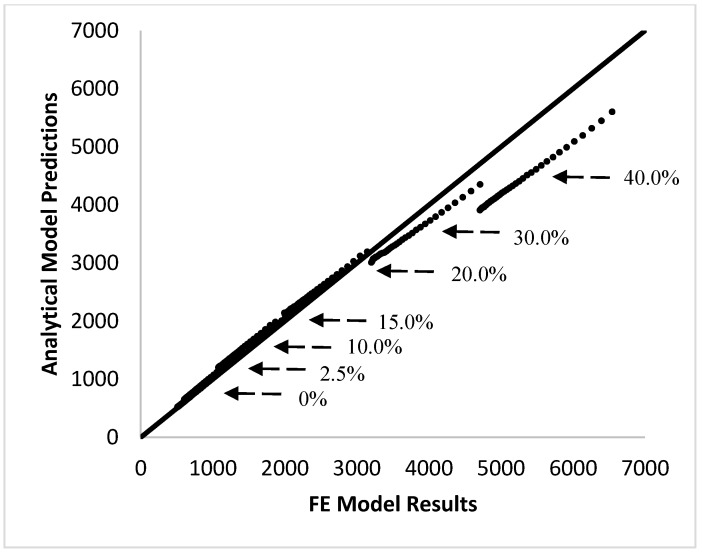
Comparison of the analytical model predictions to the finite element (FE) simulation results shows good agreement between the two at low volume fraction; however, at volume fractions equal to 30% and greater, the finite element results deviate from the predictions of the analytical model.

The accuracy of the closed-form analytical model was evaluated through comparison to the finite element experiments conducted in Abaqus CAE. The fit between the two was compared by two methods. First, Figure 2 compares the overall stress relaxation behavior as predicted by the analytical model against the data obtained through the finite element experiments. The accuracy of the model was also determined by plotting the analytical model predictions against the finite element experimental results (at the same point in time), and comparing the slope of this line to that of a y=x line. If the model is a good fit, both values should be equal and result in a slope close to one. This comparison is shown in Figure 3.

Figure 2 and Figure 3 show excellent agreement between the analytical model and the finite element model at fiber volume fractions below 30%; however, at fiber content higher than 30%, the analytical model is no longer able to accurately predict the stress relaxation behavior of the composite. This is expected because the analytical model is based on the simplified shear-lag model, which is known to be accurate only at low fiber volume fractions. Shear-lag models assume that fibers are located in an isolated pocket of resin and that the outer surface of this pocket experiences a uniform strain equal to the global strain. However, at higher volume fractions, as the fibers approach each other, the situation becomes more complex and the model is not expected to be accurate. Studies that have compared the elastic modulus of composites in a static case have shown that the shear-lag model often overpredicts the actual modulus [18].

**Figure 4:**
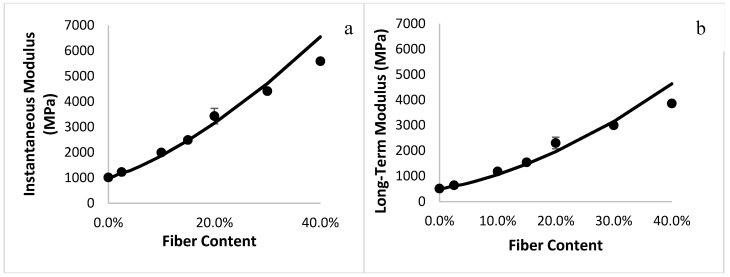
Good agreement is obtained between the instantaneous (**a**) and long-term (**b**) moduli values obtained from the analytical model (-) and the finite element simulations (●).

Figure 4 compares the analytical predictions and the results from the finite element simulation for the instantaneous and long-term moduli of the composites. An increase in the fiber content resulted in an increase in both moduli, which is a trivial effect of adding stiff glass fibers in a polymeric matrix. The results from the Monte Carlo simulations were well-predicted by the analytical model for misoriented fibers, validating the model. 

**Figure 5:**
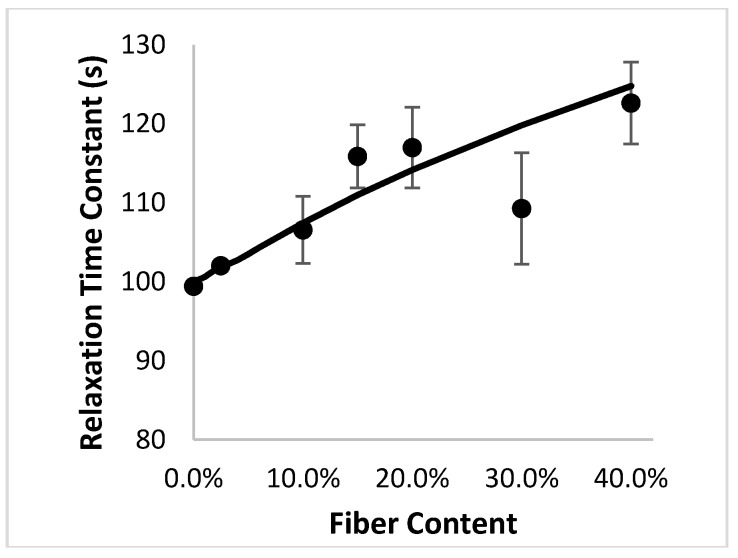
Good agreement is obtained between the relaxation rate constant obtained from the analytical model (-) and the finite element simulations (●).

Figure 5 shows the relaxation rate constant as a function of volume fraction of fibers. It is important to note that the analytical model, which is based entirely on micromechanics and makes no assertions about chemical interactions between the fiber and matrix, is able to predict a relationship between the rate constant and fiber volume fraction. The finite element results based on the Monte Carlo process are well-predicted by the analytical model for composites containing misoriented fibers. As with any experimental study, some deviations will exist between the relaxation rate constant predicted via the analytical model and those that were obtained through the finite element experiments. The Monte Carlo approach generates a set of randomized “specimens” for finite element analysis, and additional replicates could be used to futher improve the match between the two datasets. The strong agreement between the results validates the ability of the model to predict the entire stress relaxation behavior for misoriented-fiber composites at low fiber volume fractions.

**Figure 6:**
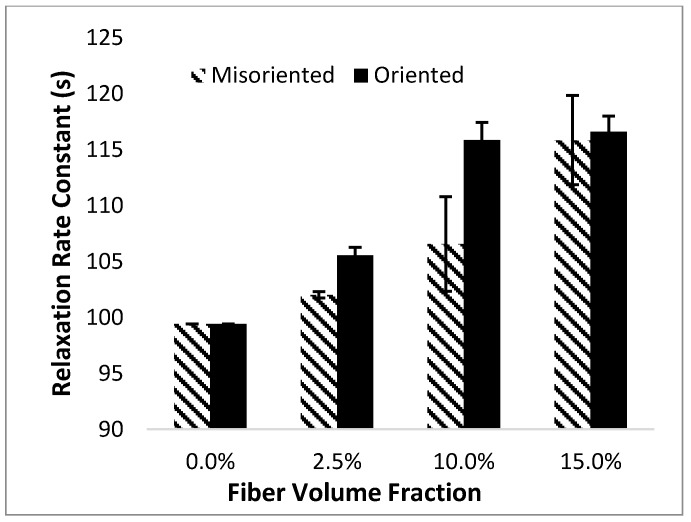
Effect of fiber orientation on the properties of the composite as obtained from finite element experiments.

In a previous study, we conducted finite element experiments on composites with oriented fibers instead of misoriented fibers; the settings used in that study were the same as those described here [12]. Figure 6 compares the effect of orientation on the results obtained from both finite element simulations. 

As mentioned earlier, the misorientation of the fibers results in a reduction in their ability to carry stress in the loading direction. Thus, for misoriented composites, the influence of the time-dependent shear lag effectiveness factor shrinks, reducing the effect of fibers on the relaxation rate constant of the composite. 

The analytical model predicts that there should be a greater difference between the relaxation time constant for oriented and misoriented samples compared to the results shown in Figure 6. However, it is important to note that the data shown in this figure was obtained through the finite element experiments and is thus subject to error due to the averaging of five replicates. Also, as mentioned earlier, the concept of a relaxation time constant depends heavily on the fit of the experimental data to a one-component Prony Series. As a result, the relaxation time constant is very sensitive to small errors in curve-fitting and variance between replicates. Although the relaxation time constant is a useful means to predict the stress relaxation rate and for comparison of overall trends, its absolute value is highly sensitive to small errors.

## 5. Conclusions

The goal of this study was to investigate the role of short fibers on stress relaxation behavior by examining the micromechanics at the fiber-matrix interface in composites with randomly oriented fibers. This novel perspective differs from previous investigations, which have focused on attributing the effect of fibers to chemical or structural changes at the interface between the fiber and the matrix. The study aimed to not only understand the role of micromechanics, but also to develop an analytical model that could be used to make predictions regarding the stress relaxation of composites with varying fiber content, orientations, and aspect ratios. 

Good agreement was observed between the results obtained via finite element experiments and the preditions from the analytical model. Thus, the analytical model developed in this study provides an adequate and accurate tool to predict the stress relaxation behavior of misoriented short-fiber composites with varying fiber content. It was found that the rate of stress relaxation is influenced by both the time-dependent elastic modulus of the matrix and the time-dependent shear stress transfer at the fiber-matrix interface, which stems from the time-dependent shear modulus of the matrix. However, the misorientation of fibers shrinks the contribution of the time-dependent shear stress transfer by a factor of one-third compared to the contribution of oriented fibers. 

The analytical model was validated using finite element experiments conducted in Abaqus CAE. Excellent agreement was observed between the analytical model and experiments at fiber volume fractions below 30%; the deviation from the analytical model beyond 30% was attributed to the inaccuracy of the shear-lag model at higher fiber volume fractions. Since the experiments followed a Monte Carlo approach with random fiber positions and orientations, its agreement with the analytical model further signifies the importance of incorporating time-dependent shear stress transfer at the fiber-matrix interface.
